# The *Non-Coding RNA* Journal Club: Highlights on Recent Papers—3

**DOI:** 10.3390/ncrna1030285

**Published:** 2015-12-21

**Authors:** Ioana Berindan-Neagoe, George Adrian Calin, Claire Francastel, Florent Hubé, Gaetano Santulli

**Affiliations:** 1 Iuliu Hatieganu University of Medicine and Pharmacy, Cluj–Napoca, Romania; E-Mail: ioananeagoe29@gmail.com; 2 M.D. Anderson Cancer Center, University of Texas, Houston, USA; 3 CNRS UMR7216, Epigenetics and Cell Fate, Université Paris Diderot, Sorbonne Paris Cité, UMR7216 Epigénétique et Destin Cellulaire, Bâtiment Lamarck B, Case Courrier 7042, 35 rue Hélène Brion, 75013 Paris, France; E-Mails: claire.francastel@univ-paris-diderot.fr (C.F.); florent.hube@univ-paris-diderot.fr (F.H.); 4 Department of Physiology and Cellular Biophysics, Clyde and Helen Wu Center for Molecular Cardiology, College of Physicians and Surgeons, Columbia University Medical Center, New York, NY 10032, USA; E-mail: gs2620@cumc.columbia.edu

## 1. Introduction

We are glad to share with you our third *Journal Club* and to highlight some of the most interesting papers published recently. We hope to help you keep aware of non-coding RNA research outside of your area. The *Non-Coding RNA* Scientific Board wishes you an exciting and fruitful read.

## 2. Noise or Sound? One Step beyond...

Highlight by Claire Francastel and Florent Hubé

Over the last few years, a growing number of studies have described neglected small open reading frames (sORF; <100aa) hidden in long non-coding RNAs (lncRNAs), using ribosome footprinting (Ribo-Seq; *i.e.*, ribosome profiling coupled to high throughput sequencing) first developed by Nick Ingolia and Jonathan Weissman. In their recent review, Muhammad Ali Mumtaz and Juan Pablo Couso provide a thorough description of the method and the type of data that one can expect through this approach [1]. In sum, Ribo-Seq allows to locate translation start sites, analyze their distribution, and measure the speed of the translating ribosomes, *i.e.*, predict elongation rate and pausing. Unexpectedly, about half of lncRNAs described so far are found associated with polysomes in mouse embryonic stem cells. Whether this association leads to the production of tiny peptides remains to be seen. However, the main conclusion of the authors and related work, “The differences between coding and noncoding RNA are likely to be blurred”, tears down one more, and maybe the ultimate, principle that distinguishes coding mRNA from “non-protein coding” ncRNAs.

## 3. At Last! All Major Classes of Small Non-Coding RNAs Gathered in a Unique Database

Highlight by Claire Francastel and Florent Hubé

Small non-coding RNAs (sncRNAs) emerged a few years ago as functionally important molecules involved in nearly all physiological and pathological processes of the cell. Numerous classes of sncRNAs have been reported that include transfer RNAs (tRNAs) and ribosomal RNAs (rRNAs) of course, but also many others like microRNAs (miRNAs) and small nucleolar (snoRNAs), the best known among them. For each class, many databases were built, some of which are now recognized references databases; this is the case for miRBase.org, which is the reference searchable database for annotated miRNAs. However, these databases are not complete since, for example, a few miRNAs annotated in UCSC were still not deposited in miRBase. Moreover, as many databases of classes of sncRNA of interest have to be assembled and curated for a given study. This drawback has now been fixed with the DASHR database from Leung *et al.* [2]. Their database accounts for about 48,000 human sncRNA genes and mature sncRNA products, and contains all major classes, including miRNAs, Piwi-interacting (piRNAs), small nuclear, nucleolar, cytoplasmic (sn-, sno-, scRNAs, respectively), tRNAs and tRNA-derived fragments, and rRNAs. Version 2.0 of the database is expected soon, but, already, seeking information in the small RNA world has never been so handy.

## 4. A New Subclass of Small Non-Coding RNA Distinct from Canonical miRNAs

Highlight by Claire Francastel and Florent Hubé

A class of small non-coding RNAs (sncRNAs), derived from mature or precursor transfer RNAs (tRNAs), are 14 to 32 nt-long single-stranded RNAs, clearly distinct from canonical microRNAs (miRNAs). There is growing evidence that these tRFs are not mere by-products of tRNAs degradation, but, instead, represent functional sncRNAs. Some tRFs are constitutively generated while others are produced in stress conditions. They appear to account for miRNAs reads in small RNA-seq data. The paper by Shigematsu and Kirino represents the first review of its kind on tRFs biogenesis and function in various organisms [3].

## 5. Micromanipulating the Behavior via microRNA: New Lessons from *Drosophila*

Highlight by Gaetano Santulli

In a very elegant paper published in Science, Joao Picao-Osorio and colleagues demonstrated that specific microRNAs (miRNA) are functionally involved in the control of behavior [4]. A mutation of a single miRNA locus (miR-iab4/iab8) affects the self-righting behavior (capacity to correct the orientation if turned upside down) of *Drosophila* larvae [4]. Small RNAs had been previously shown to affect neural differentiation, but this is the first paper to demonstrate a mechanistic role in the control of behavior. The authors also identified in the Hox gene, Ultrabithorax, one of the main miRNA targets involved in such a process. Tracking down neuronal activity using Ca2+ sensors revealed that two metameric neurons, constituting the minimal node required for self-righting behavior, display significantly different activity patterns in WT and miRNA mutants.

Since Hox gene post-transcriptional regulation has been found to be involved in self-righting control, other RNA-based regulatory processes affecting Hox gene expression may also influence neural output. These findings will most likely prompt researchers toward the study of miRNAs involved in the control of behavior in other organisms, including humans.

## 6. EMT Transition Involved in Resistance to Therapy but not in Lung Metastatic Phenotype

Highlight by Ioana Berindan Neagoe

Fischer and the group at Weill Cornell Medical College [5], demonstrated, using a complex triple transgenic mouse model (MMTV-PyMT/Rosa26-RFP/GFP/Fsp1-cre, tri-PyMT), that mice developing breast tumors in mammary gland, which metastasized in lung, conserved the epithelial features during the metastatic process, with a low population of mesenchymal cells. This observation argues for a lower involvement of epithelial-to-mesenchymal metastatic process in the lung than was initially considered, but underline a more prominent role in resistance to chemotherapy. MiR-200, a well known family of microRNA that inhibit EMT and is highly involved in the metastatic phenotype [6], was overexpressed in the proposed model, and Zeb1 and Zeb2, transcriptional repressors for E-cadherin, were not found expressed during the metastatic process. The research focus is on developing future target therapies in EMT cells [7], involved in chemoresistance, but not in metastatic phenotype acquisition.
